# Natural Variation of Seed Tocopherol Composition in Diverse World Soybean Accessions from Maturity Group 0 to VI Grown in China

**DOI:** 10.3390/plants11020206

**Published:** 2022-01-13

**Authors:** Suprio Ghosh, Shengrui Zhang, Muhammad Azam, Berhane S. Gebregziabher, Ahmed M. Abdelghany, Abdulwahab S. Shaibu, Jie Qi, Yue Feng, Kwadwo Gyapong Agyenim-Boateng, Yitian Liu, Huoyi Feng, Yecheng Li, Jing Li, Bin Li, Junming Sun

**Affiliations:** 1 The National Engineering Laboratory for Crop Molecular Breeding, MARA Key Laboratory of Soybean Biology (Beijing), Institute of Crop Sciences, Chinese Academy of Agricultural Sciences, 12 Zhongguancun South Street, Beijing 100081, China; fssuprio@gmail.com (S.G.); zhangshengrui@caas.cn (S.Z.); azaamuaf@gmail.com (M.A.); berhane76@gmail.com (B.S.G.); ahmed.abdelghany@agr.dmu.edu.eg (A.M.A.); asshuaibu.agr@buk.edu.ng (A.S.S.); qjycyz@gmail.com (J.Q.); 82101179104@caas.cn (Y.F.); k.g.agyenim.boateng@gmail.com (K.G.A.-B.); 82101201051@caas.cn (Y.L.); 82101192189@caas.cn (H.F.); 82101205019@caas.cn (Y.L.); lijing02@caas.cn (J.L.); 2 Bangladesh Agricultural Research Institute, Gazipur 1701, Bangladesh; 3 Crop Sciences Research Department, Mehoni Agricultural Research Center, Maichew 7020, Ethiopia; 4 Crop Science Department, Faculty of Agriculture, Damanhour University, Damanhour 22516, Egypt; 5 Department of Agronomy, Bayero University, Kano 700001, Nigeria

**Keywords:** tocopherols, accessions, maturity groups (MGs), geographical origin, geographical distribution, stability, soybean [*Glycine max* (L.) Merrill]

## Abstract

Tocopherols are natural antioxidants that increase the stability of fat-containing foods and are well known for their health benefits. To investigate the variation in seed tocopherol composition of soybeans from different origins, 493 soybean accessions from different countries (China, USA, Japan, and Russia) belonging to 7 maturity groups (MG 0–VI) were grown in 2 locations (Beijing and Hainan Provinces of China) for 2 years (2017 and 2018). The results showed that significant differences (*p* < 0.001) were observed among the accessions and origins for individual and total tocopherol contents. The total tocopherol content ranged from 118.92 μg g^−1^ to 344.02 μg g^−1^. Accessions from the USA had the highest average concentration of γ- and total tocopherols (152.92 and 238.21 μg g^−1^, respectively), whereas a higher level of α-tocopherol (12.82 μg g^−1^) was observed in the Russian accessions. The maturity group of the accession significantly (*p* < 0.001) influenced all tocopherol components, and higher levels of α-, γ-, and total tocopherols were observed in early maturing accessions, while late-maturing accessions exhibited higher levels of δ-tocopherol. The inclination of tocopherol concentrations with various MGs provided further evidence of the significance of MG in soybean breeding for seed tocopherol components. Furthermore, the correlation between the seed tocopherol components and geographical factors revealed that α-, γ-, and total tocopherols had significant positive correlations with latitude, while δ-tocopherol showed an opposite trend. The elite accessions with high and stable tocopherol concentrations determined could be used to develop functional foods, industrial materials, and breeding lines to improve tocopherol composition in soybean seeds.

## 1. Introduction

Soybean [*Glycine max* (L.) Merr.] seeds are consumed worldwide, owing to their nutritional values and health benefits. Soybean seeds are the principal source of natural tocopherols. Tocopherols are natural antioxidants that improve the stability of fat-containing foods and have vital biological functions as well as protect food from oxidation processes induced by free radicals [1]. They aid in preventing atherosclerosis, cancer, heart diseases, and neurological and neurodegenerative diseases such as Alzheimer’s and Parkinson’s and strengthen the immune system [2,3,4,5]. Tocopherols are classified into four types of isomers (α, β, γ, and δ), differing in their molecular structures and antioxidant activities [6]. The antioxidative activities of tocopherols in biological systems against lipid peroxidation are α > β > γ > δ (100, 50, 10, and 3% relative activity, respectively) [7]. Alpha- and γ-tocopherols are significant for their nutritional and functional effects on human health and food system stability. Delta-tocopherol shows a strong antioxidant potency in vitro [8]. Because of its nutritional value and importance for oil stability, increasing tocopherol content in soybean seeds through breeding programs has become a new and important objective. However, in order to improve soybean seed tocopherol content through conventional breeding, it is important to assess the phytochemical diversity of soybean accessions so that they can be mined for the desired traits.

Tocopherol content and composition in soybean seeds are affected by both genetic and environmental factors. High temperature and drought stress during seed maturation enhance the content of α-tocopherol in soybean seeds by two to three times, with a decrease in δ-tocopherol content [9,10]. On the other hand, low temperature (16–20 °C) during seed maturation facilitates δ-tocopherol accumulation [11] and decreases total tocopherol content in soybean seed, which is attributed to loss of γ-tocopherol [12]. The impact of environmental factors on seed tocopherol content has gotten much attention [13,14,15]. However, genotypic variability, which is the foundation of any breeding program, is seldom reported [13,16,17] for tocopherol content and profile in soybean seeds. Maturity group (MG) is an important genotypic factor influencing the quality and composition of soybean seed that has not been considered as a variable for seed tocopherol composition in previous studies. The variation in tocopherol composition across MGs and the underlying mechanisms has not yet been reported. MG, in the case of soybean, is a significant parameter that determines its geographical adaptation and facilitates the possibility of introducing new varieties, but such a crucial role in soybean breeding is still neglected. Therefore, the present investigation determined the geographical distribution of MGs and the effects of the different MGs on tocopherol content and composition of diverse world soybean accessions.

It is widely acknowledged that introducing new germplasms is essential for increasing genetic diversity and strengthening breeding stock resources. Traditional breeding techniques have been using a diverse set of germplasm to produce novel soybean lines with desirable tocopherol composition. The availability of germplasm resources plays a crucial role in the success of breeding techniques. This will allow breeders to develop new cultivars with improved tocopherol profiles and broaden soybean cultivar genetic diversity, which has been narrowed down due to the overuse of a few superior cultivars in breeding programs [18].

Germplasm collections greatly vary in their origins; how the effect of country of origin in combination with other factors such as MG, influences tocopherol composition features of soybean seeds simultaneously have not been adequately explored. In an attempt to fill this apparent gap, using similar environmental conditions, this study utilized four different soybean collections that originate from the USA, China, Russia, and Japan with varying MGs to ensure maximum sample heterogeneity. Therefore, we hypothesized that the country of origin and MG influence had a significant effect on the tocopherol composition of soybeans when they are grown in similar environments, compared to the null hypothesis that both the country of origin and MG influence did not significantly affect composition of tocopherol in soybeans.

Therefore, the current research was carried out in order to (i) investigate the variation in seed tocopherol composition and evaluate the content of tocopherols in soybean germplasms of different origins, (ii) evaluate the impact of MGs on seed tocopherol composition, (iii) visualize the geographical distribution of MG materials across different latitudes, and (iv) identify elite soybean accessions with a high and stable concentration of tocopherols across environments. Furthermore, the geographical distribution map facilitated the identification of hotspot regions representing the origin zone of the desired MGs and the selection of target MG accessions with the intended tocopherol composition. The findings of this study will open up new possibilities for the food industry and soybean breeders in terms of developing new soybean varieties with high and stable tocopherol content.

## 2. Results and Discussion

### 2.1. Variation in Seed Tocopherol Composition in Diverse Soybean Accessions

The individual and total tocopherol contents varied significantly (*p* < 0.001) among the soybean accessions (Table S1), and this revealed that genetic factors hold a crucial role in soybean tocopherol breeding. In addition, the significant differences in the individual and total tocopherol contents between years (Table S1) suggested that seasonal variabilities as evident from the weather information could also affect tocopherol production in soybean seeds. The lack of significant year × accession interactions for all traits except total tocopherol indicates that despite the seasonal variabilities, the accessions with high and low tocopherol maintained their trend for *α-*, *γ-,* and *δ-* tocopherols. The total tocopherol levels ranged from 118.92 μg g^−1^ to 344.02 μg g^−1^, with a mean of 229.12 μg g^−1^ (Table 1). The highest total tocopherol content was observed in accession PI592523 (344.02 μg g^−1^) from the USA, probably due to the presence of comparatively higher content of γ-tocopherol in the USA soybean accessions (assuming that γ-tocopherol accounts for 60% to 70% of the total tocopherol) [2,19,20] and the lowest was observed in WDD02708 (118.92 μg g^−1^) from Russia (Table S2), representing a nearly threefold difference between both two accessions. 

For the individual tocopherols, the α-tocopherol levels ranged from 3.15 μg g^−1^ to 33.38 μg g^−1^ with an average of 10.84 μg g^−1^ (Table 1). The highest level of α-tocopherol was observed in the accession ZDD00041 (33.38 μg g^−1^) from China, whereas ZDD12828 (3.15 μg g^−1^) from China had the lowest level, indicating a more than 10-fold difference between the highest and lowest-containing accessions. This finding is consistent with earlier research [13,16]. Accession ZDD00041 from China belongs to MG 0 (Table S3) and had the highest level of α-tocopherol, originating in the high latitude Northern Region (NR), which represents hotspots for the origin of accessions with increased α-tocopherol accumulation [21]. Moreover, α-tocopherol showed the highest coefficient of variation (CV) (42.04%) (Table 1), implying that α-tocopherol exhibits a more natural variation across environments than other tocopherol isomers, which is also supported by previous studies [13,22]. 

The γ-tocopherol levels ranged from 79.31 μg g^−1^ to 220.91 μg g^−1^ with a mean of 143.15 μg g^−1^ (Table 1). The highest level of γ-tocopherol was observed in the accession PI592523 (220.91 μg g^−1^) from the USA, which may be attributed to the presence of γ-tocopherol as the most prevalent form of vitamin E in the US diet [23,24], and the lowest was recorded in WDD02958 (79.31 μg g^−1^) from Russia (Table S2). Therefore, accession PI592523 from the USA was typically notable for containing the highest amount of γ- and total tocopherol and could be an important cultivar in nutraceuticals and functional food sectors. 

Similarly, δ-tocopherol levels ranged from 21.87 μg g^−1^ to 136.67 μg g^−1^ with an average of 75.11 μg g^−1^ (Table 1). The lowest content of δ-tocopherol was obtained in the accession WDD02708 (21.87 μg g^−1^) from Russia, perhaps due to the presence of low δ-tocopherol in the accessions of high-latitude regions [21], and the highest content was found in ZDD06638 (136.67 μg g^−1^) from China (Table S2). In the current study, the proportional percentages of α-, γ-, and δ-tocopherols were 4.73%, 62.47%, and 32.78%, respectively, across environments and genotypes (Table 1), which were within the same range as the values reported previously [10,13,25]. Such wide variations in levels of tocopherols will offer possibilities of developing superior accessions to produce high-quality soybean products such as edible oil, soymilk, tofu, soy paste, soy sauce, and natto.

### 2.2. Germplasm Origins Had a Significant Influence on Seed Tocopherol Composition

**Figure 1:**
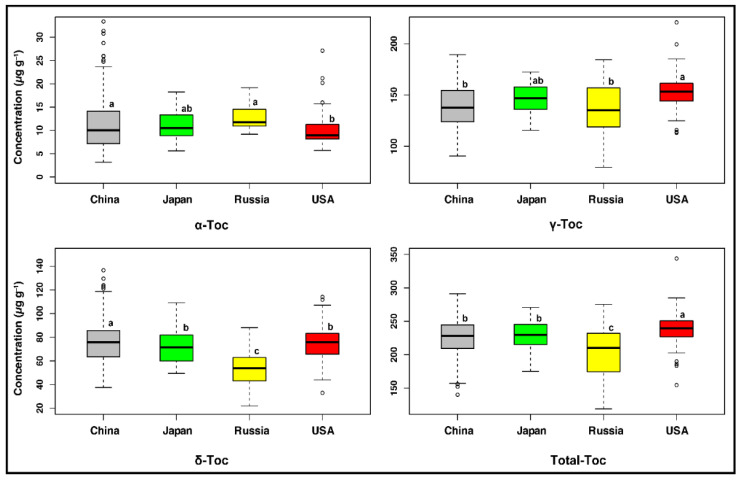
Variations in individual and total tocopherol levels among soybean accessions collected from the USA, China, Japan, and Russia (averaged across two locations and two years). The lines across the box plot indicate the medians. Different lowercase letters (a, b, and c) represent statistically significant differences at the *p* < 0.05 level. Values followed by the same letter are not significantly different at the *p* < 0.05 level. Sample size for China = 326, Japan = 25, Russia = 19, and USA = 123. Here, Toc represents tocopherol.

We observed highly significant variations (*p* < 0.001) among the germplasm origins for α-, γ-, δ-, and total tocopherol contents (Table S1), which was in agreement with a previous study [26]. Specifically, there were significant differences (*p* < 0.05) observed between Chinese and USA soybean accessions in terms of individual and total tocopherol contents (Figure 1). Meanwhile, year by origin interaction did not show significant effects on the individual and total tocopherol contents (Table S1), indicating that genetic factors have a greater impact on tocopherol variation in soybean seeds than environmental factors, which was consistent with a previous report [27].

The USA accessions had the highest average concentration of total tocopherol (238.21 μg g^−1^), followed by the Japanese (228.50 μg g^−1^) and Chinese accessions (227.22 μg g^−1^), whereas the Russian accessions revealed the lowest total tocopherol content (203.58 μg g^−1^) (Figure 1). The total tocopherol content did not differ significantly between the Japanese and Chinese accessions, which was consistent with Song et al. [28], implying that the proximity of these two origins may have led to a lack of significant effect on soybean quality components.

For the individual tocopherols, the Russian accessions had the highest mean content of α-tocopherol (12.82 μg g^−1^), followed by the Chinese (11.07 μg g^−1^) and Japanese accessions (10.91 μg g^−1^), while the lowest level of α-tocopherol (9.94 μg g^−1^) was found in the USA accessions, which differed significantly from the other three germplasm origins (Figure 1). In the current study, the USA accessions had the lowest mean content of α-tocopherol and may be attributed as part of the reasons for a lower intake of α-tocopherol by US adults [29]. In contrast, the Russian accessions had the highest mean content of α-tocopherol due to the accumulation of increased α-tocopherol by the accessions that originated in the higher latitude regions [21].

In contrast to the content of α-tocopherol, the USA accessions had the highest mean concentration of γ-tocopherol (152.92 μg g^−1^), followed by the Japanese (145.81 μg g^−1^) and Chinese accessions (139.64 μg g^−1^), while the lowest γ-tocopherol (136.75 μg g^−1^) was recorded from the Russian accessions (Figure 1). The USA accessions had the highest mean content of γ-tocopherol because around 70% of the vitamin E consumed in the typical US diet comes from γ-tocopherol [20]. It could be due to the higher utilization of soybean, sesame, and corn oil in the US diet [24].

The Chinese accessions displayed the maximum mean content of δ-tocopherol (76.52 μg g^−1^), followed by the USA (75.35 μg g^−1^) and Japanese accessions (71.78 μg g^−1^), while the Russian accessions contained the lowest mean δ-tocopherol level (54.01 μg g^−1^) (Figure 1). The Russian accessions showed the highest mean content of α-tocopherol (12.82 μg g^−1^) and the lowest mean content of δ-tocopherol (54.01 μg g^−1^). This demonstrates the possibility of decreasing δ-tocopherol levels with increasing α-tocopherol levels. Such an inverse connection between α- and δ-tocopherols was also evident in numerous previous studies [11,17,21,22].

The Russian and Chinese accessions exhibited no significant differences in α- and γ-tocopherol contents due to the closeness of the Heilongjiang province of China and the far-eastern region of Russia, which are the central regions for soybean production in China and Russia and share similar MGs [30]. Overall, the Chinese accessions displayed significantly greater diversity for α- and δ-tocopherols, whereas the USA accessions demonstrated greater diversity for γ- and total tocopherols across environments.

### 2.3. Maturity Groups Had a Pronounced Impact on Seed Tocopherol Composition

The analysis of variance revealed significant variations (*p* < 0.001) for individual and total tocopherol contents among different maturity groups (Table S1), which may be attributed to the length of time required for soybean vegetative and reproductive development affected by the maturity group. Earlier studies have also found that MG significantly impacts soybean seed quality, such as protein, oil, fatty acid, isoflavone, soluble sugar, etc., which is decided by the day length and temperature during the crop growing period [10,31,32,33,34]. The significant variations in individual and total tocopherol levels between years (Table S1) revealed that seasonal variabilities significantly affect the tocopherol profile of soybean accessions from different MGs. Therefore, we should consider the effect of MG and select the stable tocopherol components in multiple environments and years for the soybean breeding program.

**Figure 2:**
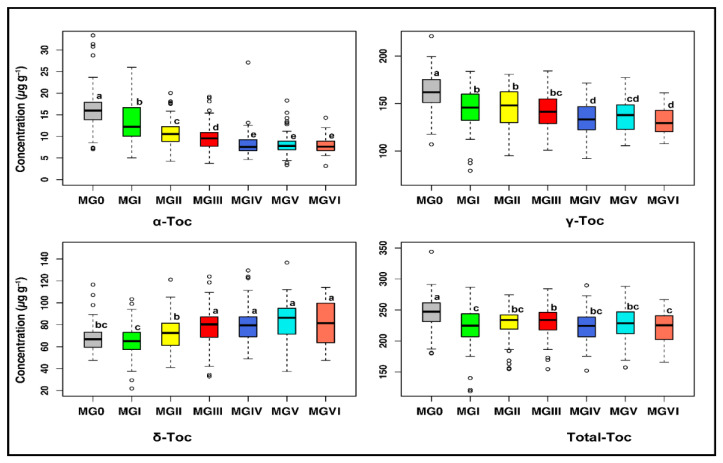
Variability of tocopherol contents among the accessions of different maturity groups (MGs). Box plots were used for identifying outliers and for comparing distributions between different maturity groups. Different lowercase letters (a, b, c, d, and e) represent statistically significant differences at the *p* < 0.05 level. Values followed by the same letter are not significantly different at the *p* < 0.05 level. Sample size for MG 0 = 68, MG I = 83, MG II = 79, MG III = 96, MG IV = 82, MG V = 54, and MG VI = 31. Here, Toc represents tocopherol.

All individual tocopherols showed significant associations (*p* < 0.01) with maturity groups (Figure S1). The levels of α- and γ-tocopherols showed a negative and significant linear association with MG (*r* = −0.93** and *r* = −0.90**, respectively) (Figure S1A,B). The means of α-, γ-, and total tocopherol ranged from 7.95–16.03 μg g^−1^, 131.32–161.67 μg g^−1^, and 220.97–245.99 μg g^−1^ in MG 0–VI, respectively (Figure 2 and Table S4). There were 2.01-fold and 1.23-fold higher α-tocopherol and γ-tocopherol contents in MG 0 than MG VI, respectively. For total tocopherol, there was a similar tendency to that observed in α- and γ-tocopherol levels but no significant association. The level of total tocopherol ranged from 220.97 μg g^−1^ in MG VI to 245.99 μg g^−1^ in MG 0 (Table S4). Therefore, the higher levels of α-, γ-, and total tocopherols were presented in the earlier maturity groups, while the lower levels were observed in the later maturity groups. In contrast, the δ-tocopherol level revealed a positive and significant linear relationship with MG (*r* = 0.91**) (Figure S1C), indicating that there was a higher level of δ-tocopherol observed in the later maturity groups. MG V had a 1.27-fold δ-tocopherol level than MG I (Table S4). These findings reflect contrasting trends of tocopherols with different MGs and further emphasize the significance of MGs as a determining factor in soybean seed tocopherol composition. Early maturing soybean accessions had higher levels of α-, γ-, and total tocopherols due to the exposure of early MG genotypes to long day length and higher solar radiation conditions at high latitude region during seed filling period (R5–R8 stage), which leads to higher tocopherol levels [35,36,37] and due to origin and distribution of early MG accessions in the high-latitude regions [21]. High-latitude regions (NR of China, Far-East region of Russia, and Northern USA) were not considered suitable for the production of late MG genotypes because they exhibit short frost-free periods that are not long enough for the maturation of late-maturing accessions. Late maturing accessions had a higher level of δ-tocopherol due to exposure of late MG genotypes in cooler environments during the seed filling period [11] and due to origin and distribution of late MG accessions in the low-latitude regions (SR of China, Southeast Asia, and Southern USA) [21,38]. Furthermore, the findings showed that early MGs could affect soybean seed tocopherol composition differently comparing to late MGs at the same planting site or even on the same planting date, which could be due to the differences in growth periods of both early and late-maturing accessions. Because α-tocopherol exhibits the highest vitamin E activity [3,39], this result suggested that soybean accessions from early MGs would be more advantageous for producing high-quality tocopherols than accessions from late MGs. Thus, the results indicate that breeders should pay much more attention to soybean maturity groups when producing varieties for a particular trait of interest [31].

### 2.4. Principal Component Analysis (PCA) Based on Origin and Maturity Groups

**Figure 3:**
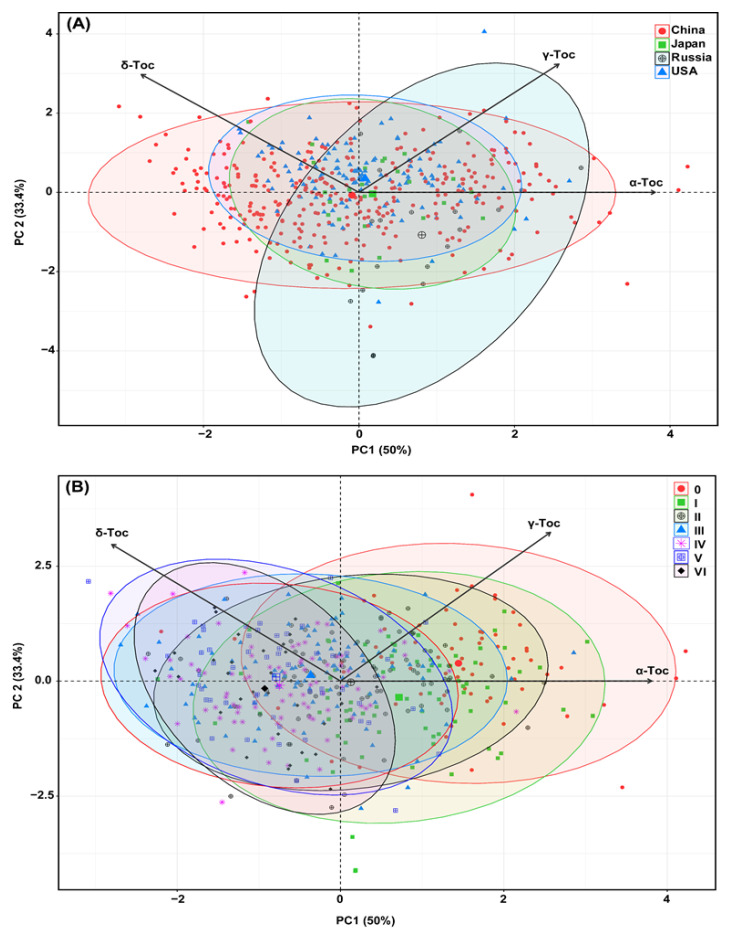
Principal component analysis (PCA) biplots of soybean seed tocopherol compositions of 493 diverse soybean accessions averaged across all environments (**A**) PCA for country of origin; (**B**) PCA for maturity group (MG). Each of the points on the biplot represents a single accession; the accessions are color-coded with different symbols that signify their origin and maturity group. Here, Toc represents tocopherol.

PCA is a clustering technique commonly used to discover variables between populations of multiple categories, identify key influencing parameters, and reveal interactions between parameters [40]. The application of PCA is one of the methods breeders use to detect influential features for appropriate selection in cultivar development. Here, two PCAs were created based on the origin (Figure 3A) and MGs (Figure 3B). Both PCAs demonstrated a greater diversity for origin and MGs, which were similar to the boxplot results (Figure 1 and Figure 2).

The PCA results revealed that the first two components (PC1 and PC2) accounted for 83.4% of the total variance in tocopherol compositions. The first axis (PC1) explains 50% of the total variance, and the principal contributor to the variance in PC1 was α-tocopherol (50.10%), followed by δ-tocopherol (27.08%) and γ-tocopherol (22.81%). The second axis (PC2) explains 33.4% of the total variance, and most of the variance was by γ-tocopherol (54.23%), followed by δ-tocopherol (45.76%), while α-tocopherol had the lowest contribution to PC2 variability (0.00002%). From the biplot, δ-tocopherol is inversely correlated with α-tocopherol; such a contrasting relationship has been observed previously [22]. The origin biplot (Figure 3A) demonstrated that most of the Chinese accessions were largely dispersed over all tocopherol isomers except γ-tocopherol, elucidating the minimal level of the γ-tocopherol present in the Chinese soybean accessions. The majority of the accessions from the USA were clustered around γ-tocopherol variables, suggesting that accessions of the USA contain higher levels of γ-tocopherol. In addition, accessions from Russia were laid closer to the α-tocopherol variable, implying their tendency for higher performance of such components. The biplot of the MGs (Figure 3B) revealed that most of the accessions from MG 0–II were scattered around the α- and γ-tocopherols, indicating that early maturing accessions retain high levels of α- and γ-tocopherols. Furthermore, the dispersion of accessions from MG IV–VI across the δ-tocopherol variables revealed their tendency to have higher contents of δ-tocopherol.

### 2.5. Geographical Distribution of Soybean Seed Tocopherol Components

Length of growing period or maturity is a vital feature of crops since it influences the geographical adaptation of a variety [41]. Soybean cultivars’ growth cycles (from sowing/emergence to the reproductive stage) vary in different ecological regions. As a result, the content of tocopherols varies greatly among soybean varieties worldwide. Tocopherol components are affected by geographic variables (latitude, longitude, and altitude) (Table S5) in different soybean accessions, indicating that the origin of the accessions has a significant impact on the composition of tocopherols. Therefore, the mean individual and total tocopherol contents of accessions across locations and years are significantly associated with geographical factors such as the latitude, longitude, and altitude of their corresponding region of origins (Table S5). Longitude and latitude have a direct impact on the environment and climate. However, latitude has a more significant impact on climate than longitude due to the apparent rise in temperatures near the world’s equator.

In this study, α-, γ-, and total tocopherols showed significant positive associations with latitude (*r* = 0.66***, 0.49***, and 0.13**, respectively) (Table S5), indicating that the contents of α-, γ-, and total tocopherols were relatively higher in accessions from higher latitude regions, which is consistent with our previous report [21]. This may be due to the growth period of soybean accessions of high latitude regions being shorter than those planted at low latitude regions, which introduces the grain filling period to long day length and higher solar radiation conditions, therefore, facilitating the accumulation of more α- and γ-tocopherols in the seeds of high latitude accessions. In addition, higher-latitude regions are the zones of adaptation for soybean MGs ranging from 0 to II [32,42,43], which characterizes the situation of early-maturing accessions that contain higher levels of α-, γ-, and total tocopherols. The geographical distribution map of MGs also revealed a similar pattern (Figure S2).

On the other hand, δ-tocopherol showed significant and negative correlations with latitude (*r* = −0.56***) (Table S5), reflecting that lower latitude accessions produce higher levels of δ-tocopherol due to exposure of late-maturing accessions to cooler environments during the grain filling period [11]. Lower-latitude regions are the areas of adaptation for the late-maturing accessions (MG IV to VI), which was also evident from the geographical distribution map (Figure S2). These findings are consistent with our earlier research [21]. However, the geographical distribution map of MGs (Figure S2) clearly showed that early MGs were distributed in the higher-latitude regions and late MGs were distributed in the lower-latitude regions, which also indirectly illustrated the association between tocopherol content and geographical factors.

Moreover, we also observed that α-, γ-, and total tocopherol level of soybean accessions planting in Hainan were significantly higher than in Beijing (Table S6). This consequence indicates that high air temperature during seed filling period in Hainan (R5-R8 stages, 25–28 °C of mean temperature on March in Hainan, Table S7) can accelerate the accumulation of α-, γ-, and total tocopherols in soybean seeds, which is consistent with the previous results [9,15]. Therefore, it can be concluded that the level of α-, γ-, and total tocopherol contents of the Northern Region (NR) accessions becomes significantly higher when cultivated in the Southern Region (SR) under higher temperature environments during their seed filling period. However, no significant difference was observed for δ-tocopherol content (Table S6).

### 2.6. Stability Performance of Soybean Accessions for Tocopherols across Environments

**Figure 4:**
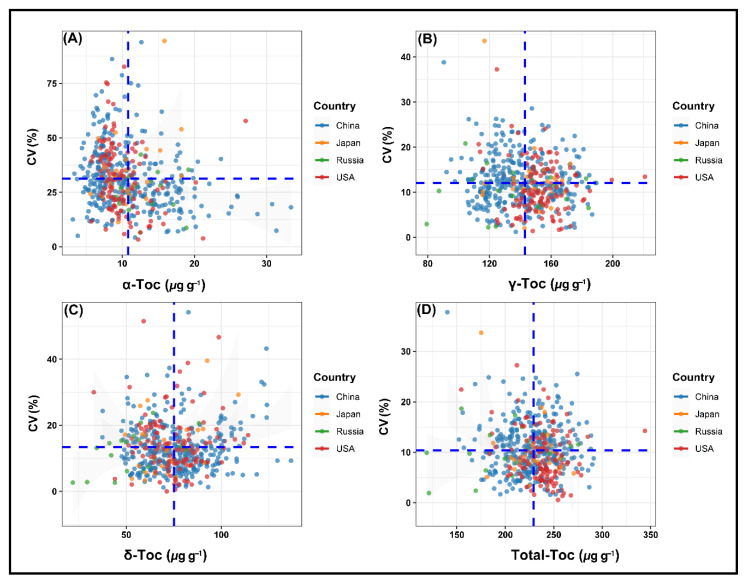
Scatter plots showing the relationship between means of tocopherol components and coefficient of variation (CV) for 493 soybean accessions grown in four different environments: (**A**) scatter plot of α-tocopherol; (**B**) scatter plot of γ-tocopherol; (**C**) scatter plot of δ-tocopherol; (**D**) scatter plot of total tocopherol. Horizontal and vertical dashed lines, in blue, represent the average CV and mean of the tocopherols, respectively.

Soybeans are cultivated across a broad latitude range (about 20 to 53° N) under a wide range of environmental circumstances in China [44]. The stability of soybean accessions varies significantly under different environmental conditions [45]. The CV of each accession was used to reflect the stability of accession, as a lower CV indicates higher stability for a cultivar in various environments. Soybean accessions that maintain high levels of desired tocopherols with high stability for various ecoregions would be useful for genetic improvement, varietal introduction, and soybean breeding programs aimed at improving soybean tocopherol components. Since the accumulation of tocopherols is significantly affected by planting locations [27]; therefore, it is important to identify stable and high tocopherol-containing soybean genotypes among different environments. Based on CV, the top five high tocopherol containing stable genotypes from different origins are listed in Table 2. These genotypes can be recommended due to their overall performance. The means of the seed tocopherols were plotted against their CVs to show how much the optimal level of each tocopherol was stable within each germplasm origin (Figure 4A–D).

Accessions having a higher content of α-tocopherol and lower CV values (Figure 4A) are more preferred because α-tocopherol is the most active form of vitamin E in the biological system of humans [8] because of its strong affinity for the hepatic tocopherol transfer protein [3,39]. Therefore, the main interest among tocopherols remains in α-tocopherol. Since soybean seeds contain a low concentration of α-tocopherol, hence, vitamin E activity in soybean is lower than that of other oilseed crops [3]. Thus, increasing the α-tocopherol content and enhancing the vitamin E activity in soybean seed is an important breeding objective, which could contribute in reducing the high production cost of synthetic α-tocopherol. In the present study, we identified novel soybean varieties with high α-tocopherol contents. Among all soybean accessions, ZDD00041 from China had higher α-tocopherol content (33.38 μg g^−1^) with lower CV (18.14%), followed by another Chinese accession ZDD23615, as its α-tocopherol content was 31.35 μg g^−1^ with a lower CV of 7.42%. Other accessions with higher and stable α-tocopherol content are presented in Table 2. Therefore, these accessions could be used as a breeding parent to produce soybean cultivars with high levels of α-tocopherol that open up new market opportunities for soybeans.

A collection of the USA, Chinese, Japanese, and Russian accessions with high γ-tocopherol content are shown in Figure 4B. The accessions that revealed higher stability and higher γ-tocopherol levels are critical for soybean crops to inhibit oil peroxidation and enhance oil quality [46]. The USA accession PI592523 showed higher γ-tocopherol content (220.91 μg g^−1^) followed by another USA accession WDD01715 (199.44 μg g^−1^), but their CV values (13.45% and 12.72%, respectively) were higher than the average CV values (11.96%) of γ-tocopherol, indicating that these genotypes were less stable (Figure 4B). The results showed that among soybean accessions, ZDD06815 from China had higher γ-tocopherol content (186.69 μg g^−1^) with lower CV (5.08%), followed by WDD02019 from the USA, which had a higher γ-tocopherol content (185.16 μg g^−1^) with a lower CV of 10.32%. Other stable accessions with higher γ-tocopherol levels are shown in Table 2.

Accessions with a higher level of δ-tocopherol and lower CV values (Figure 4C) are also one of the desired forms, since δ-tocopherol is more effective than α- or γ-tocopherol in suppressing tumor growth [47]. The Chinese accession ZDD06638 showed the highest δ-tocopherol content of 136.67 μg g^−1^ with a CV of 9.29%, followed by ZDD06595 from China, which had a higher δ-tocopherol content (129.54 μg g^−1^) with a lower CV of 9.30%. Other accessions with higher δ-tocopherol levels demonstrating greater stability are listed in Table 2.

Total tocopherol content was calculated by summing up the contents of individual isomers; hence, increasing the total tocopherol content is one of the most effective ways to enhance the amount of a particular type of tocopherol isomer. Therefore, accessions that reveal higher stability and higher total tocopherol levels are also vital for soybean breeders. For total tocopherol, accession PI592523 from the USA showed the highest mean total tocopherol content (344.03 μg g^−1^), but it seems unstable due to a higher CV value (14.25%) (Figure 4D). Based on the current results, the Chinese accession, ZDD24336, was the most suitable and desirable genotype, which showed a stable (CV: 8.80%) total tocopherol level (291.24 μg g^−1^) at different sites (Table 2). Other accessions with higher total tocopherol levels exhibiting greater stability are shown in Table 2. This implied that soybean accessions mentioned in Table 2 are suited for the production of high and stable concentrations of tocopherols across environments that could be used for industrial and pharmaceutical applications and genetic improvement of soybean tocopherols. Our results (Table 2) showed that low latitude (SR of China) cultivars were more susceptible to light and temperature than high latitude (NR of China) cultivars, suggesting that high latitude cultivars have broader regional adaptability than low latitude cultivars, which is associated with the large CV values of the Southern Region (SR) soybean accessions. This finding was in agreement with earlier studies [48,49,50]. Taken together, it is concluded that introducing new germplasms to China will provide promising germplasm resources with desired tocopherol profiles and excellent stability over a wide range of environments [51].

Soybean is a short-day plant that is extremely sensitive to photoperiods [52]. This high sensitivity to photoperiods severely hampers the improvement of soybean. Moreover, tocopherol content in soybean seed is substantially affected by photoperiods. Thus, soybean accessions from the four origins with stable tocopherol content in different environments are thought to be insensitive to the day length or photoperiod, which will aid in the genetic improvement of soybean tocopherol composition with wide adaptability. The findings of this research will contribute greatly to the selection of accessions with optimum tocopherol levels, which are less affected by varying environmental conditions. The identified soybean accessions could be exploited as elite genetic materials for soybean breeding and tocopherol industries.

## 3. Materials and Methods

### 3.1. Chemicals and Reagents

Standards of α-, γ-, and δ-tocopherols purchased from Sigma-Aldrich Chemical Company (Sigma Aldrich Co., St. Louis, MO, USA) were used for the quantification of tocopherol components. High-Performance Liquid Chromatography (HPLC) grade ethanol (Thermo Fisher Scientific, New Brunswick, NJ, USA) and methanol (Thermo Fisher Scientific, Ottawa, ON, Canada) were used as an extraction solvent and mobile phase, respectively. All other chemicals used in the present study were of analytical grade.

### 3.2. Plant Materials

A total of 493 soybean accessions, including 326 Chinese, 123 USA, 25 Japanese, and 19 Russian accessions covering 7 MGs from MG 0–VI, were employed in this investigation. The accessions were chosen based on their availability at the soybean genetic resource research group of the Institute of Crop Sciences (ICS), Chinese Academy of Agricultural Sciences (CAAS). The accessions were divided into distinct MGs as MG 0 (68 accessions), MG I (83 accessions), MG II (79 accessions), MG III (96 accessions), MG IV (82 accessions), MG V (54 accessions), and MG VI (31 accessions). MG III contained the maximum number of accessions that were collected from various locations. Accession number, accession name, origin, and MG of all accessions are listed in Table S3. Among the whole collection, Chinese accessions were obtained from the three principal soybean-growing regions, namely Northern Region (NR), HuangHuaiHai Valley Region (HR), and Southern Region (SR) of China, covering a geographical range from 19.1° N to 53° N [44]. The ranges of MGs for the NR, HR, and SR are MG 000–II, II–V, and IV–VIII, respectively [43]. For the USA accessions, the selected 133 accessions covered various MGs from MG 0–VI. Along with the Chinese and American accessions, a few Russian and Japanese accessions were also examined to provide a more diversified panel of accessions.

### 3.3. Field Experiments

Field experiments were carried out in two different locations: Changping, Beijing (40°13′ N and 116°12′ E), and Sanya, Hainan (18°24′ N and 109°5′ E) in 2017 and 2018. The accessions were planted on 12 June 2017 and 14 June 2018 in Changping and 14 November 2017 and 16 November 2018 in Sanya. The locations differ significantly in climate. The monthly temperature (°C), precipitation (mm), and sunshine (h) readings for the experimental sites in Hainan and Beijing are presented in Table S7. Soil total nitrogen, phosphorus, and potassium levels were 80.5 mg kg^−1^, 68.7 mg kg^−1^, and 12.31 g kg^−1^, respectively, at Changping and 98.59 mg kg^−1^, 39.68 mg kg^−1^, and 80.78 g kg^−1^, respectively, at Sanya. The experimental details, as well as plot management operations, have already been reported [53]. Briefly, the experiment was laid out in a randomized incomplete block design, with the two planting locations used as replications. At each location, separate randomization was performed, and seeds of each accession were sown in a single row plot of 3 m in length with 0.5 m spacing between rows and 0.1 m between plants within rows. After emergence, plants were thinned to leave only uniform healthy plants. During land preparation, organic fertilizer was applied at the rate of 15 t ha^−1^, which contained 30 kg N ha^−1^, 60 kg P ha^−1^, and 50 kg K ha^−1^. Weeds were controlled by postemergence application of 2.55 L ha^−1^ of acetochlor, as well as hand weeding during the growing season. When plants reached physiological maturity, plots were harvested manually. After harvesting, all the seeds of each accession were pooled together, and about 100 seeds were randomly picked from each accession to determine tocopherol levels.

### 3.4. Tocopherol Extraction and Determination

Tocopherol components were detected from matured soybean seeds using a reverse-phase HPLC (Agilent Technologies, Santa Clara, CA, USA) system following the method described by Dwiyanti et al. (2007) [54] with some modifications. The detailed procedure used for tocopherol determination in the present study has been previously reported [21]. Tocopherols were quantified using standard curves calculated by the linear regression analysis. The sum of each tocopherol’s content was used to compute the total tocopherol content. The content was calculated as μg of tocopherol g^−1^ of the soybean seed sample in fresh weight.

### 3.5. Statistical Analysis

Descriptive statistics were primarily used to interpret the data for each tocopherol isomer across environments. Minimum, maximum, mean, standard deviation (Std.), and coefficient of variation (CV) of individual and total tocopherol contents were estimated using Excel software (Microsoft Office Professional Plus 2013, Santa Rosa, CA, USA). Analysis of variance (ANOVA) for seed tocopherol composition was performed using the general linear model technique (PROC GLM) in SAS 9.2 [55]. Three separate ANOVAs were calculated to evaluate the effect of accessions, country of origin, and MGs on seed tocopherol content. Accessions, origin, and MGs were treated as fixed effects in each ANOVA. Whereas the different planting locations were employed as replications and together with year, they were regarded as a random effect. Principal component analysis (PCA), Pearson’s correlation coefficients (*r*), scatter plots, and boxplots were analyzed and visualized using R statistical software version 3.5.0 (R Foundation for Statistical Computing, Vienna, Austria). Scatter plots were utilized to show the association between different MGs and tocopherols and the relationship between the mean of each tocopherol component and its CV. Differences were considered significant in all cases when the *P*-value was less than 0.05 (*p* < 0.05). The discriminative tocopherol components in soybean seeds were visualized and identified using PCA. The correlation analysis was carried out between the components of seed tocopherol and geographical variables (latitude, longitude, and altitude). A geographical distribution map of world soybean MGs was developed with ArcGIS 10.0 (ESRI, Redlands, CA, USA, http://desktop.arcgis.com/en/arcmap/, accessed on 9 December 2021) using ordinary kriging interpolation. ArcGIS is extensively used in geographic information system (GIS) technologies since it can be effectively used to create maps, compile geographical data, and preserve spatial data.

## 4. Conclusions and Future Perspectives

The variation observed in tocopherol composition among four worldwide germplasm accessions may provide elite genetic resources for soybean breeding. The accessions from the USA had the highest average concentration of γ- and total tocopherols, whereas a higher level of α-tocopherol was observed in the Russian accessions, which illustrates the influence of ecoregions on individual and total tocopherol contents of soybean accessions. This study may also provide relevant information to consumers and food sectors when evaluating soybean accessions from different MGs in terms of tocopherol content. To the best of our knowledge, the present study is the first to address the response of soybean tocopherols to MGs where it was indicated that higher levels of α-, γ-, and total tocopherols were observed in early maturing accessions, while higher levels of δ-tocopherol could be obtained from cultivating late-maturing accessions. MGs are useful in evaluating this chemical component, which mainly determines the shelf life and flavor of soybean oil. Furthermore, the geographical map can be used to identify hotspot regions where MGs possess greater levels of desired tocopherol components. Overall, the soybean accessions with high stability and desirable tocopherol content identified in this study could be useful for consumption and cultivar development.

These findings would help make decisions early in the selection of elite soybean with desired levels of both individual and total tocopherols. This may encourage researchers to develop future investigations for further molecular and metabolic research works to validate the usefulness of this bioactive chemical component in improving human feeding.

## Figures and Tables

**Table 1:** Variations in tocopherol contents among all soybean accessions grown in two different locations for two years (2017 and 2018).

Tocopherol Isomers	Minimum (μg g^−1^)	Maximum (μg g^−1^)	Mean (μg g^−1^)	Std.	CV (%)
*α*-Tocopherol	3.15	33.38	10.84	4.56	42.04
*γ*-Tocopherol	79.31	220.91	143.15	20.64	14.41
*δ*-Tocopherol	21.87	136.67	75.11	16.90	22.50
Total-Tocopherol	118.92	344.02	229.12	27.22	11.88

Std. = standard deviation. CV = coefficient of variation.

**Table 2:** List of soybean accessions that exhibit desired contents of tocopherols with higher stability (lower CV).

Tocopherol Isomers	ID Number	Name	Mean (μg g^−1^)	CV (%)	Origin
Higher α-Toc	ZDD00041	Heihe1	33.38	18.14	China
	ZDD23615	Hefeng47	31.35	7.42	China
	ZDD22657	Hefeng35	30.77	19.59	China
	ZDD24346	Kenfeng22	28.74	15.01	China
	ZDD22798	Dongnong42E	25.99	22.89	China
Higher γ-Toc	ZDD06815	Heihe4	186.69	5.08	China
	WDD02019	9234	185.16	10.32	USA
	ZDD24376	Hefeng52	184.33	11.14	China
	WDD02599	C∏691	184.33	6.52	Russia
	ZDD24342	Kenfeng13	183.86	7.37	China
Higher δ-Toc	ZDD06638	Baishuidou	136.67	9.29	China
	ZDD06595	Maojihui	129.54	9.30	China
	ZDD06361	Dapudou	118.63	5.14	China
	ZDD13149	Qingpidou	111.35	4.95	China
	ZDD14232	Huangdou	108.15	9.02	China
Higher Tot-Toc	ZDD24336	Huajiang4	291.24	8.80	China
	ZDD24157	Quxian1	289.75	7.61	China
	ZDD01629	Liaodou26	286.65	3.08	China
	ZDD06815	Heihe4	285.45	6.43	China
	ZDD24685	Jidou17	279.22	6.73	China

Here, Tot-Toc represents total tocopherol. CV = coefficient of variation.

## Data Availability

Data is contained within the article and Supplementary Materials.

## Supplementary Materials

The following are available online at https://www.mdpi.com/article/10.3390/plants11020206/s1. Figure S1: Relationship between means of soybean seed tocopherol composition (μg g^−1^) with maturity groups (MG 0–VI), Figure S2: Geographical distribution of 493 maturity group (MG) materials in different geographic regions of the world, Table S1: Analysis of variance (ANOVA) showing the effect of accession, country of origin and maturity group (MG) on the tocopherol content of soybean accessions grown at two locations of China for two years, Table S2: List of soybean accessions with highest and lowest contents of tocopherols, Table S3: Information of the 493 accessions used in this study, Table S4: Tocopherol composition (μg g^−1^) in soybean seeds from different maturity groups (MGs) across two years (2017 and 2018), Table S5: Correlations between soybean tocopherol isomers and geographical factors, Table S6: Variation in individual and total tocopherol contents of soybean accessions planted in two different locations for two years (2017 and 2018), Table S7: Monthly temperature (°C), precipitation (mm), and sunshine (h) readings at the two experimental regions in China in 2017 and 2018.
