# Supplementary material for: Natural Variation of Seed Tocopherol Composition in Diverse World Soybean Accessions from Maturity Group 0 to VI Grown in China
Source: Plants (Basel). 2022 Jan 13;11(2):206. doi: 10.3390/plants11020206 (PMC8779575; doi:10.3390/plants11020206)

Figure S1. Relationship between means of soybean seed tocopherol composition (µg g^{-1}) with maturity groups (MG 0–VI). (A) α-Toc, (B) γ-Toc, (C) δ-Toc, and (D) Total-Toc. Here, Toc represents tocopherol.

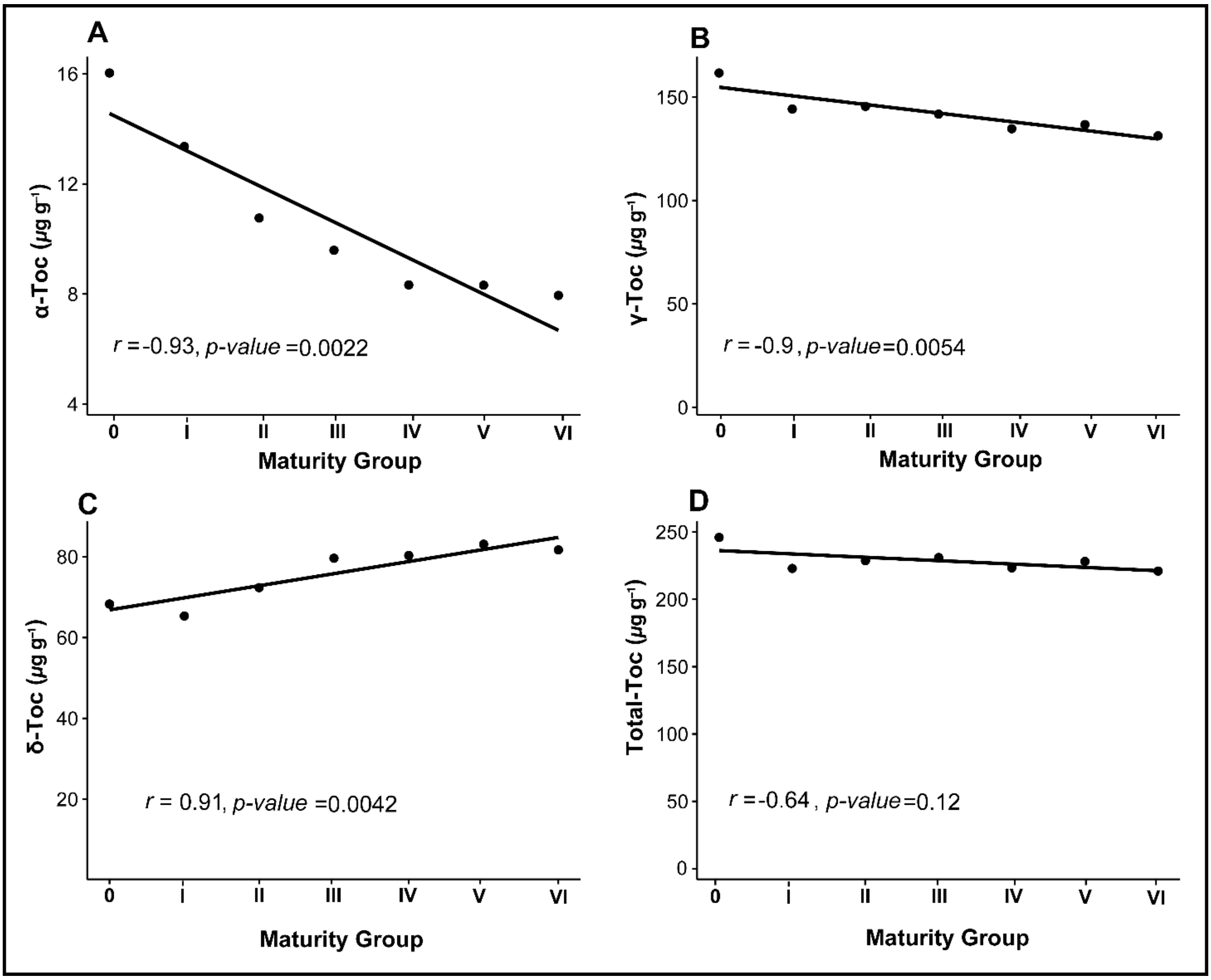

Figure S2. Geographical distribution of 493 maturity group (MG) materials in different geographic regions of the world. Here, NC, Northern China; HC, HuangHuaiHai China; SC, Southern China; FERU, Far-East of Russia; CRU, Central Russia; NUSA, Northern United States of America and SUSA, Southern United States of America. The different colour of dots represents different MG types of 493 accessions in 8 geographic regions. The maturity group materials are mapped according to their region of origin.

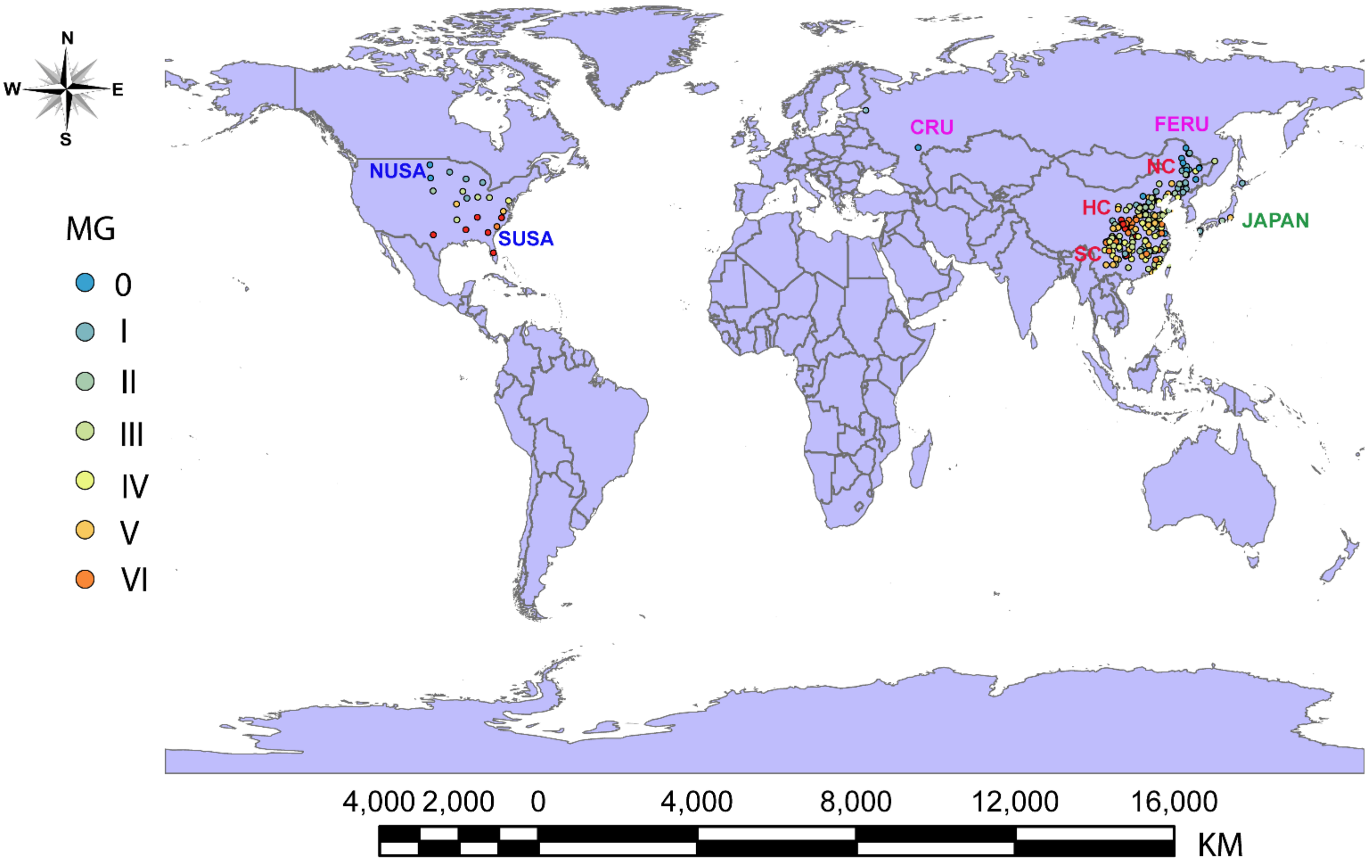

Supplement: Supplementary file 1 [file plants-11-00206-s001.zip › Figure S1-S2.pdf]
