# Supplementary material for: Natural Variation of Seed Tocopherol Composition in Diverse World Soybean Accessions from Maturity Group 0 to VI Grown in China
Source: Plants (Basel). 2022 Jan 13;11(2):206. doi: 10.3390/plants11020206 (PMC8779575; doi:10.3390/plants11020206)
Supplement: Supplementary file 1 [file plants-11-00206-s001.zip › Table S1-S2.pdf]

**Table S1.** Analysis of variance (ANOVA) showing the effect of accession, country of origin and maturity group (MG) on the tocopherol content of soybean accessions grown at two locations of China for two years (separate ANOVA was performed for accession, origin, and maturity group)

| **Variation source** | **Tocopherol Contents** | | | |
|---|---|---|---|---|
| | **α-Tocopherol** | **γ-Tocopherol** | **δ-Tocopherol** | **Total-Tocopherol** |
| **Year** | *** | *** | *** | *** |
| **Accession** | *** | *** | *** | *** |
| **Year × Accession** | NS | NS | NS | * |
| | | | | |
| **Year** | *** | *** | *** | *** |
| **Country of Origin** | *** | *** | *** | *** |
| **Year × Country of Origin** | NS | NS | NS | NS |
| | | | | |
| **Year** | *** | *** | *** | *** |
| **MG** | *** | *** | *** | *** |
| **Year × MG** | NS | ** | NS | *** |

Here, *, ** and *** represent the significance levels at $p < 0.05$, $p < 0.01$, and $p < 0.001$, respectively; NS = non-significant

**Table S2.** List of soybean accessions with highest and lowest contents of tocopherols

| Tocopherols | ID Number | Name | Mean (μg $g^{-1}$) | Origin |
|---|---|---|---|---|
| α-Tocopherol | ZDD12828 | SNFTJSD | 3.15 | China |
| | ZDD00041 | Heihe1 | 33.38 | China |
| γ-Tocopherol | WDD02958 | POLUKULTURNAYA-2 | 79.31 | Russia |
| | PI592523 | Glacier | 220.91 | USA |
| δ-Tocopherol | WDD02708 | PSB313 | 21.87 | Russia |
| | ZDD06638 | Baishuidou | 136.67 | China |
| Total-Tocopherol | WDD02708 | PSB313 | 118.92 | Russia |
| | PI592523 | Glacier | 344.02 | USA |
