# Supplementary material for: Natural Variation of Seed Tocopherol Composition in Diverse World Soybean Accessions from Maturity Group 0 to VI Grown in China
Source: Plants (Basel). 2022 Jan 13;11(2):206. doi: 10.3390/plants11020206 (PMC8779575; doi:10.3390/plants11020206)
Supplement: Supplementary file 1 [file plants-11-00206-s001.zip › Table S4-S7.pdf]

Table S4. Tocopherol composition (μg g^{-1}) in soybean seeds from different maturity groups (MGs) across two years (2017 and 2018)

MG		α-Tocopherol	γ-Tocopherol	δ-Tocopherol	Total-Tocopherol
0	Mean	16.03 a	161.67 a	68.29 bc	245.99 a
	Range	7.02-33.38	107.08-220.91	47.48-116.59	179.90-344.02
I	Mean	13.36 b	144.24 b	65.33 c	222.93 c
	Range	5.03-25.99	79.31-183.86	21.87-103.35	118.92-286.65
II	Mean	10.76 c	145.54 b	72.36 b	228.66 bc
	Range	4.24-20.04	95.12-180.71	41.08-121.19	154.68-274.47
III	Mean	9.59 d	141.79 bc	79.63 a	231.02 b
	Range	3.74-19.17	100.91-184.33	32.93-124.01	154.57-284.07
IV	Mean	8.33 e	134.71 d	80.32 a	223.36 bc
	Range	4.65-27.09	92.17-171.58	48.93-129.54	152.18-289.74
V	Mean	8.32 e	136.72 cd	83.10 a	228.13 bc
	Range	3.37-18.3	105.66-177.37	37.44-136.66	157.28-288.14
VI	Mean	7.95 e	131.32 d	81.70 a	220.97 c
	Range	3.15-14.31	107.86-161.32	47.48-114.12	165.68-266.84

Here, values of means within each column with different letters indicate statistically significant differences at $p < 0.05$.

Table S5. Correlations between soybean tocopherol isomers and geographical factors [a]

**Geographical factor** [b]	**α-Tocopherol**	**γ-Tocopherol**	**δ-Tocopherol**	**Total-Tocopherol**
**Latitude**	0.66***	0.49***	–0.56***	0.13**
**Longitude**	0.13**	–0.25***	–0.016	–0.18***
**Altitude**	–0.14**	–0.0097	–0.053	–0.063

[a] Values are represented as Pearson's correlation coefficients (*r*). ** and *** are significant at $p < 0.01$ and $p < 0.001$, respectively. Values without asterisks are not significant at $p < 0.05$.

[b] Geographical factors (latitude, longitude, and altitude) data for the geographical origins of accessions were used in the correlation analysis.

Table S6. Variation in individual and total tocopherol contents of soybean accessions planted in two different locations for two years (2017 and 2018)

Location	α-Tocopherol (μg g^{-1})	γ-Tocopherol (μg g^{-1})	δ-Tocopherol (μg g^{-1})	Total-Tocopherol (μg g^{-1})
**Beijing**	8.82b	134.98b	76.34NS	220.15b
**Hainan**	12.66a	150.65a	74.17NS	237.49a

Different lowercase letters (a and b) demonstrate statistically significant differences at the $p < 0.05$ level; NS = non-significant

Table S7. Monthly temperature (°C), precipitation (mm), and sunshine (h) readings at the two experimental regions in China in 2017 and 2018

**Hainan, Sanya**												
Month	October		November		December		January		February		March	
Year	2017	2018	2017	2018	2017	2018	2017	2018	2017	2018	2017	2018
Max. (°C)	29	28	28	24	25	22	24	23	24	24	27	34
Mean (°C)	28	24	26	21	23	18	22	19	22	19	25	28
Min. (°C)	25	21	23	20	20	16	19	16	19	15	22	24
Rainfall (mm)	262.7	95.4	79.3	79.8	21.8	18.6	40.1	16.2	33.6	16	124.9	22.3
Sunshine (h)	166.5	202.5	136	90.5	132.5	155.5	131	124.5	138	173.5	200	245
**Beijing, Changping**												
Month	June		July		August		September		October		November	
Year	2017	2018	2017	2018	2017	2018	2017	2018	2017	2018	2017	2018
Max. (°C)	30	33	37	33	36	32	32	26	20	21	11	14
Mean (°C)	28	28	35	29	34	28	29	21	18	19	9	11
Min. (°C)	23	22	31	25	30	24	24	17	15	15	6	8
Rainfall (mm)	67	35.5	69.5	127	135.8	57.2	8.2	19	84.4	3.7	0	2.7
Sunshine (h)	323.5	350.5	336	303	314	329	325	316	173.5	219	215	207.5
